# IMFLKD: an incentive mechanism for decentralized federated learning based on knowledge distillation

**DOI:** 10.1038/s41598-026-46234-1

**Published:** 2026-03-30

**Authors:** Xukai Ying, Keyang Yan, Xizhang Gao, Jie Huang

**Affiliations:** 1 Department of Computer Science, Zhejiang University of Science and Technology, Hangzhou, 310023 China; 2 School of Arts and Sciences, Rutgers University, New Brunswick, 08901 USA; 3 State Key Laboratory of Resources and Environmental Information Systems, Institute of Geographic Sciences and Natural Resources Research, Beijing, 100101 China

**Keywords:** Federated learning, Knowledge distillation, Blockchain, Incentive mechanism, Engineering, Mathematics and computing

## Abstract

Knowledge Distillation-based Federated Learning (KD-FL) has garnered significant attention as one of the core technical pathways for next-generation Federated Learning (FL), owing to its communication efficiency, privacy preservation, and strong robustness. Meanwhile, to further reduce reliance on a central server, blockchain-enabled KD-FL architectures have become a research hotspot. However, designing an effective incentive mechanism that encourages participants to consistently contribute high-quality knowledge remains a fundamental challenge for ensuring the system’s long-term sustainability. To address this issue, this paper proposes an Incentive Mechanism for decentralized FL based on Knowledge Distillation (IMFLKD). First, we design a two-stage evaluation method, combining smart contract-based label aggregation and peer-wise comparison, that enables accurate client model quality estimation and fair reward allocation without increasing time complexity. Second, we establish a multi-dimensional dynamic reputation system based on the Subjective Logic model, incorporating metrics such as data quality, activity level, and stability to identify high-value participants and incentivize sustained, high-quality contributions across multiple FL rounds rather than short-term opportunistic behavior. Finally, we integrate these components into a decentralized, blockchain-enabled KD-FL framework. Experimental results demonstrate that IMFLKD achieves superior performance in contribution assessment accuracy, computational overhead, and resilience against malicious attacks, showcasing strong practicality and reliability.

## Introduction

With data becoming the core driver of artificial intelligence, sharing and utilizing data while preserving privacy has emerged as a critical challenge. Federated learning (FL), as a distributed machine learning paradigm, enables model training without requiring raw data to leave local devices, making it one of the key technologies for secure data sharing^1^. However, traditional FL architectures based on a star topology introduce risks of single points of failure and potential abuse of centralized authority, making them unsuitable for application scenarios that require equal relationships among participants or large-scale network deployments^2^. Recently, an increasing number of researchers have begun to explore blockchain-enabled FL frameworks^3^. Blockchain, as a decentralized distributed ledger, offers features such as transparency, immutability, and traceability, making it an ideal infrastructure for FL. It can serve both as a secure medium for data storage and as a decentralized model aggregator, effectively addressing the vulnerability of the central server to failure and the trust issues inherent in conventional FL systems^4^.

The simple replacement of the central server in FL with a blockchain does not sufficiently guaranty the privacy of the participants or the security of the global model and introduces a series of new challenges^5^. In blockchain-enabled systems, all participants can access training data, such as gradient updates, uploaded to the chain. However, researchers have demonstrated that it is possible to reconstruct the original private data of a client merely from updates to its local model in multiple rounds of federated training^6^. Therefore, a naive integration of blockchain and FL fails to effectively defend against inference attacks or Byzantine behaviors from other nodes. Moreover, parameter files of complex models are relatively large, storing all clients’ model parameters on-chain at every training round incurs substantial storage overhead^7^.

To further enhance data privacy, researchers have proposed Knowledge Distillation (KD)-based FL (KD-FL)^8^. As an effective model aggregation approach, KD-FL significantly reduces communication overhead by exchanging soft labels instead of model parameters, thereby addressing the challenges of model compression and collaborative optimization under resource constraints in distributed settings^9^. The core mechanism of KD-FL revolves around knowledge transfer and privacy preservation: within the federated framework, each participant (e.g., mobile devices, edge nodes, or institutional servers) trains a lightweight student model locally, and aligns its knowledge using soft labels provided either by a central server or a global teacher model, without directly sharing raw data or model parameters.

However, in the KD-FL, designing a well-structured incentive mechanism is crucial to encourage participants to consistently and reliably contribute high-quality knowledge, such as soft labels, features, or model outputs. Due to heterogeneous data distributions across clients, their contributions are inherently uneven. If rewards are allocated unfairly based on actual contributions, participant motivation will be severely undermined, significantly hindering the broader adoption and development of KD-FL^10^. Fair FL^11^ has emerged as a key enabler for fostering healthy collaboration and building a sustainable FL ecosystem, as it ensures that clients’ interests are protected and that they receive equitable treatment. In recent years, numerous Fair FL approaches have been proposed, aiming to achieve fairness throughout the entire federated training pipeline–from client selection and contribution evaluation to incentive allocation. Among these components, contribution evaluation plays an indispensable role, as it provides the critical basis for both client selection and reward distribution^12^. Specifically, high-performing clients are typically selected based on their evaluated contributions to enhance global model performance. Moreover, many incentive mechanisms are explicitly designed around clients’ contribution scores. Therefore, fairly assessing each client’s contribution to the global model’s performance has become a central research challenge in Fair FL^13^.

This study addresses the lack of a fair and efficient incentive mechanism in KD-FL by tightly integrating it with blockchain technology, and proposes a decentralized incentive mechanism. The main contributions of this paper are as follows:

- We propose an Incentive Mechanism for decentralized FL based on Knowledge Distillation (IMFLKD). First, we design a two-stage evaluation framework–label aggregation followed by peer comparison–implemented via smart contracts, which enables estimation of client model quality and fair reward allocation without increasing time complexity. Second, we establish a multi-dimensional dynamic reputation system based on the Subjective Logic model, incorporating metrics such as data quality, activity level, and behavioral stability to identify and prioritize high-quality participants. This approach incentivizes clients to consistently contribute high-quality knowledge across multiple FL rounds, rather than engaging in opportunistic short-term behavior. Such a long-term incentive mechanism enhances the overall stability and effectiveness of the system.
- We design a blockchain-enabled decentralized KD-FL framework, which replaces the centralized server in traditional FL with blockchain technology, thereby enhancing system transparency and security. Moreover, by incorporating KD-FL, where participants exchange soft labels instead of model parameters, we significantly reduce communication overhead. To address the lack of a public proxy dataset, we propose a data-free KD-FL method based on Conditional Variational Autoencoders (CVAE), which generates a high-quality “pseudo-public dataset” to enable KD in scenarios where no shared public data is available. Additionally, we integrate a negative distillation defense module, which not only improves model accuracy but also strengthens the system’s resilience against malicious attacks.

## Literature review

### Knowledge distillation-based FL

To overcome the limitations of traditional FL, researchers introduced KD into the FL framework, leading to the development of KD-FL, which has since become a mainstream approach for addressing heterogeneous FL^14^. Early KD-FL approaches relied on a shared, unlabeled public dataset to align the output spaces and provide a unified knowledge representation for heterogeneous models. The extended federated distillation method FedAUX was proposed to leverage an unlabeled auxiliary dataset for unsupervised pre-training and weighted integrated client models^15^. Li Hu et al. proposed MHAT, a model-heterogenous aggregation training scheme for FL, utilizes KD to extract the update information and trains an auxiliary model on the server^16^. Moreover, many works such as FedKD^17^, FedAD^18^, and DS-FL^19^, assume the existence of a public proxy dataset.

The public data needs to be similar in distribution to the local data, otherwise it can easily cause domain shift. To reduce dependence on public datasets, researchers have turned to generative models to synthesize knowledge carriers^20^. Qi et al. proposed a FL framework based on Bidirectional KD named FedBKD, which generated a public dataset by CVAE without sharing the private data^21^. Several data-free KD-FL methods have also been proposed, such as FedKFD^22^, which focuses on knowledge validation, and FedAlign^23^, which trains the generator on the server without accessing local data. Furthermore, recent data-free methods have further optimized issues such as parameter transmission-free^24^ and challenges like data distribution heterogeneity^25^, making them applicable to large-scale recommendation models and personalized FL. However, public-data-free methods rely on the quality of generative models and incur high training complexity.

To address issues in KD-FL such as single-point failure of the central server, communication bottlenecks, and security concerns, researchers have introduced decentralized architectures^26^. Witt et al. proposed a blockchain-enabled FL framework that integrates KD with 1-bit compressed soft labels, uses smart contracts for aggregation, and introduces a peer truth serum for federated distillation incentive mechanism to realize a lightweight decentralized framework^27^. Deng et al. proposed a blockchain-enabled federated KD framework BFKD and a practical Byzantine fault-tolerant algorithm for federated score grouping to improve consensus efficiency, with high data mining performance and communication efficiency^28^. Research shows that decentralized architectures not only enhance the system’s robustness and privacy, but also unleash the collaborative potential of edge devices^29^ .

Despite its rapid development, KD-FL still faces the challenge of quantifying clients’ knowledge contributions. This difficulty enables free-riding or the submission of low-quality knowledge by some clients, thereby degrading the overall system efficiency. To address this issue, this study adopts a decentralized architecture that leverages blockchain’s immutability, traceability, and the automated execution capability of smart contracts to provide a trusted, decentralized computing environment for the proposed KD-FL incentive mechanism in this paper.

### Incentive mechanism in FL

In real-world scenarios, participants in FL systems often have heterogeneous computational resources and varying data quality^30^. Without an effective incentive mechanism, it would be difficult for participants to sustainably and consistently contribute high-quality efforts.

Contribution-based incentive mechanisms are a core strategy in FL incentive design^31^. Early work primarily drew on the Shapley value from cooperative game theory, which computes the change in model utility caused by a participant’s inclusion or exclusion to theoretically achieve fair contribution allocation^32^. However, the exponential computational complexity of the Shapley value makes it infeasible for large-scale FL systems. Researchers have therefore proposed various approximation methods, such as FedShapleX^33^, WTDP-Shapley^34^, and GTG-Shapley^35^, which significantly reduce computational overhead while maintaining evaluation accuracy. In recent years, contribution evaluation has been further integrated with specific FL tasks. Lei et al. proposed FedDSV, a contribution estimation framework, compatible with the Shapley value fairness properties in dynamic scenarios where participants randomly join or leave, and introduced a Monte Carlo variant sampling method to reduce computational complexity^36^. Liu et al. proposed FairFed, which treats FL as multiple single-stage cooperative games, and introduce cooperative Shapley value to ensure fairness, and leverage strategic equivalence to reduce computation from exponential to polynomial complexity^37^. However, contribution-based incentive mechanisms still face challenges in adaptability to dynamic environments, robustness, and fairness.

To mitigate risks such as free-riding, low-quality updates, and even malicious attacks, reputation-based incentive mechanisms have been introduced^38^. Their core idea is to maintain a dynamically updated reputation score for each client, which comprehensively reflects the reliability, consistency, and cooperativeness of its historical behavior, and to determine the client’s future participation eligibility, aggregation weight, or resource allocation priority accordingly^39^. Xin Chang et al. proposed DriveFL, a dynamic reputation-based incentive mechanism for FL in dense vehicular networks^40^ . This mechanism quantifies quality assessment records and integrates reverse auction theory to attract vehicles with higher data quality, thereby improving model training quality under constraints of communication cost and budget. Hongyun Cai et al. proposed an incentive mechanism based on two-way reputation, which introduces a reputation blockchain to prevent malicious nodes from tampering with reputation scores and to defend against collusion attacks between edge servers and devices. The mechanism dynamically provides rewards according to data quality^41^. However, such mechanisms still face challenges including the cold-start problem, insufficient adaptability to dynamic environments, and lazy behavior of participants^42^.

Therefore, our research comprehensively integrates contribution-based and reputation-based incentive mechanisms, constructs a lightweight contribution evaluation framework, incorporates multi-dimensional reputation modeling, and establishes a cross-round long-term trust system to maintain the stability and effectiveness of the incentive mechanism.

## Methods

### Framework

**Fig. 1:**
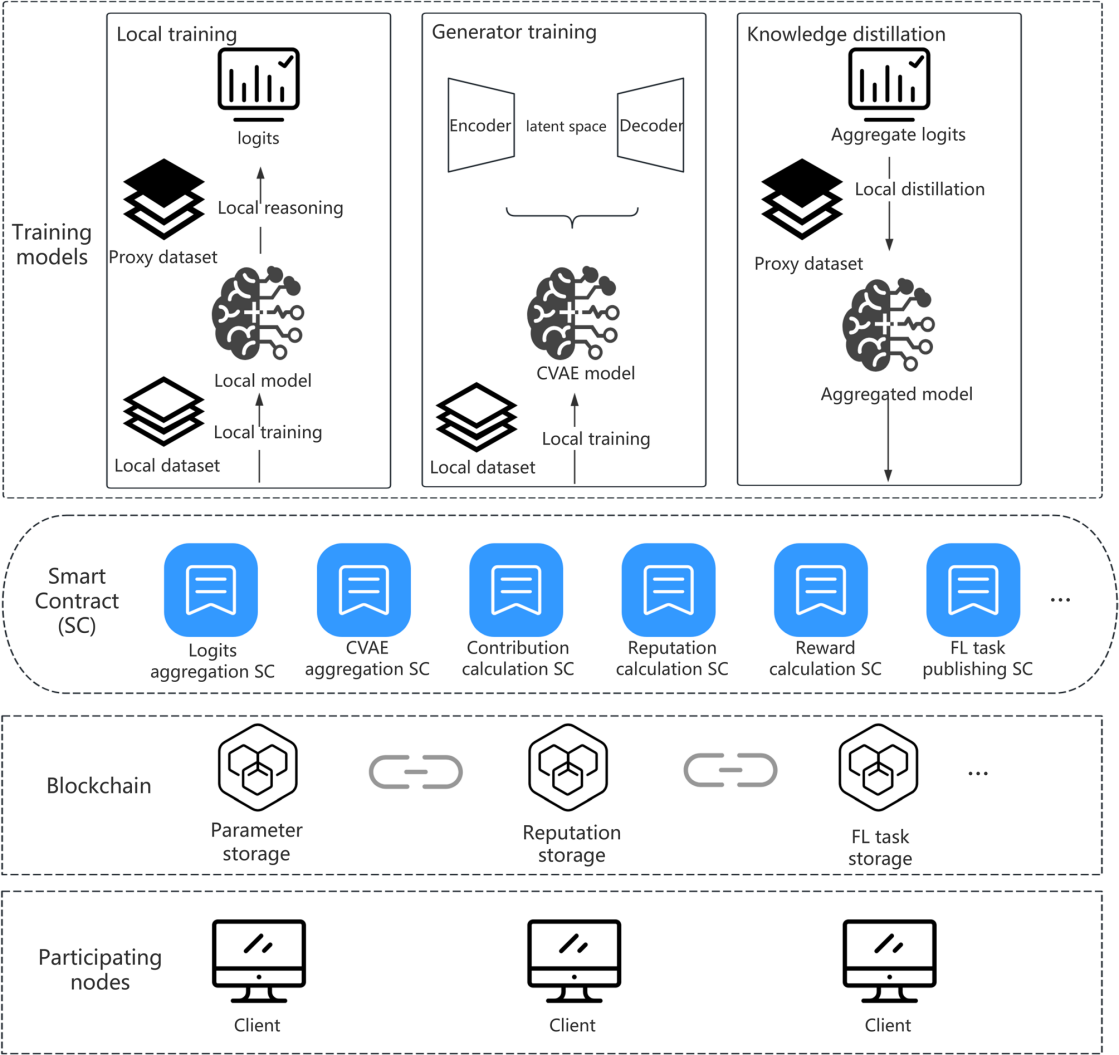
Decentralized KD-FL system architecture.

First, we designed a blockchain-enabled decentralized KD-FL framework, in which the behavior and contributions of each node in the system are recorded on the distributed ledger of the blockchain, laying the foundation for subsequent incentive mechanism design and trust management. As shown in Fig. 1, the framework mainly consists of four parts:

1. Participating nodes. Each participating node $i \in \{1,2,\ldots ,N\}$ takes part in federated training, holding a local dataset $D_i$, and trains its local model $\theta _i^{(t)}$ in each round *t*. Additionally, participating nodes also engage in the blockchain consensus process, responsible for validating and generating blocks that contain model update information.
2. Blockchain. Replaces the centralized server in traditional approaches, providing notarization and coordination for the FL process. To ensure security and efficiency in the FL scenarios, several improvements have been made to the underlying blockchain mechanism: (a) participants must obtain identity authentication from the task initiator in advance; (b) the training process remains relatively private within the consortium blockchain; and (c) the transaction fee mechanism is eliminated, and rewards are instead distributed based on participants’ contributions.
3. Smart contracts. Smart contracts are deployed on the blockchain and execute automatically when predefined conditions are met. In this system, the smart contracts are responsible for initializing the system state, assigning initial reputation scores, and dynamically maintaining participants’ reputation values and access permissions to the global model during execution, thereby ensuring fairness and traceability of the system.
4. Training models. The system involves two types of training models: the local models maintained by each participating node and the global model aggregated through KD-FL. Each participating node performs local training to minimize a loss function defined over its private dataset:
  1 $$\begin{aligned} \min _{\theta _i} F_i(\theta _i) := \sum _{(x_i^k,y_i^k)\in D_i} L_i(h_{\theta _i}(x_i^k),y_i^k) \end{aligned}$$
  where $L_i$ is the loss function of the model on the data sample $(x_i^k,y_i^k) \in D_i$. The goal of honest participants is to collaboratively train a high-performing global model without disclosing their private data.

Based on the above architecture, each participant is responsible for submitting prediction results on a proxy dataset using their locally trained model. The smart contract replaces the central server, handling the aggregation of participants’ predictions, computing their contributions, and distributing rewards accordingly.

Each participant trains a CVAE^43^ model $\theta _i$ on their private dataset $X_i^{\text {priv}}$, optimizing both the reconstruction loss and the KL divergence to obtain the encoder and decoder parameters. Simultaneously, they compute and record their local label distribution $Y_i^{\text {dist}}$. Each participant then uploads the decoder parameters $\omega _i$ and their local label distribution $Y_i^{\text {dist}}$ to the blockchain. The smart contract aggregates the uploaded decoder parameters to construct a global decoder $\bar{\omega }$, and fuses the local label distributions into a global label distribution $\bar{Y}^{\text {dist}}$. The FL initiator then uses the aggregated decoder $\bar{\omega }$ to sample in the latent space and generates a “pseudo-public dataset” $X_{\text {pub}}$ according to the global label distribution $\bar{Y}^{\text {dist}}$, which is subsequently distributed to all participants.

Each participant trains a primary task model $\theta _i$ on their private dataset $X_i^{\text {priv}}$, and then performs inference on the public dataset $X_{\text {pub}}$ to generate soft-label outputs $Y_i$. These prediction results $Y_i$ are uploaded to the blockchain. The smart contract computes a global soft label $\bar{Y}_{\text {pub}}$ by aggregating the uploaded predictions using a weighted strategy based on participants’ reputation scores, thereby providing a unified “teacher signal” for KD. Each participant downloads the aggregated soft labels $\bar{Y}_{\text {pub}}$ and uses the public dataset $X_{\text {pub}}$ to perform KD on their local model, enabling knowledge fusion and performance improvement.

### Workflow of the IMFLKD

**Fig. 2:**
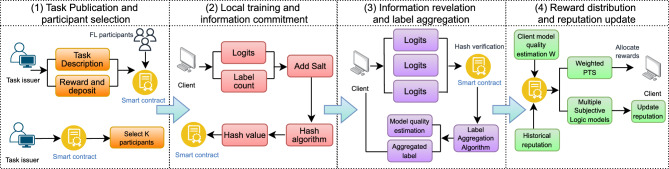
Workflow of our proposed IMFLKD.

Based on the decentralized KD-FL architecture constructed above, our proposed IMFLKD accurately evaluates the contribution of participants through label aggregation techniques, implements fair reward distribution using an improved peer truth serum algorithm, and constructs a dynamic reputation system combined with a multi-dimensional Subjective Logic model, thereby incentivizing high-quality participation. The IMFLKD process is automatically executed through smart contracts. As shown in Fig. 2, the entire process is mainly divided into four stages:

1. Task publication and participant selection. The task issuer publishes a FL task, specifying the task requirements, and reward criteria. A smart contract filters eligible participants based on the task characteristics and their historical reputation. Participants respond to the task invitation by submitting resource commitments and prepare to enter the FL process.
2. Local training and information commitment. Participants train local models based on their private datasets and generate predictions (logits) on a public dataset. They apply salted hashing to the prediction results and label statistics, then submit the hash values to the smart contract. The smart contract records all participants’ hash commitments, providing a verification benchmark for the subsequent revelation phase.
3. Information revelation and label aggregation. After all participants have completed their commitments, the smart contract initiates the revelation phase. Participants disclose their prediction results and random salt values. The smart contract verifies whether the revealed information matches the previously submitted hash values, executes the label aggregation algorithm, and estimates the true label distribution and participant quality scores based on the participants’ prediction results.
4. Reward distribution and reputation update. Based on the label aggregation results, the quality scores and contribution weights of each participant are calculated. The weighted peer truth serum algorithm is executed to compute the reward distribution according to the participants’ contribution levels and peer consensus. The participants’ reputation values are updated using a multi-dimensional Subjective Logic model, and the updated results are recorded on the blockchain.

### Contribution evaluation based on label aggregation

Accurately evaluating the contribution of participants is the core issue in the design of incentive mechanisms. By employing label aggregation techniques to estimate soft labels from the participants’ predictions, and then quantifying each participant’s contribution by comparing their predictions with this soft label, their contribution can be effectively measured.

The contribution evaluation method we designed is inspired by the LAONEPASS algorithm proposed by Yang et al.^44^. Through dynamic Bayesian network modeling, it can efficiently estimate participant quality and true labels with up to 2 iterations, eliminating the need for explicit termination conditions. We apply this algorithm for the first time in the field of KD-FL. By modeling the prediction results uploaded by participants, we can estimate both the model quality and the distribution of true labels in one iteration. The quality evaluation results are directly used for subsequent reward allocation.

We model the label aggregation problem as a dynamic system, where each task corresponds to a time slice $t \in \{1,2,\ldots ,T\}$. The system includes the following key variables:

- *M*: Total number of participants
- $w_t^i$: Quality of participant *i* at time slice *t*(dynamic variable)
- $x_{i,t}$: Predicted label of participant *i* for task *t*(observed variable)
- $y_t$: True label of task *t*(latent variable)

The structure of the dynamic Bayesian network includes two types of edges:

1. Inter-time-slice edge: Connect variables within the same time slice, $w_t^i$, $x_{i,t}$ and $y_t$.
2. Intra-time-slice edge: Connect quality variables of participants across different time slices, reflecting the temporal evolution of quality.

Based on the dynamic Bayesian network modeling, we design a KD-FL label aggregation algorithm. The algorithm achieves online learning by iteratively updating participant quality and true label estimation.

At each time slice *t*, the quality estimation formula for participant *i* is:

2 $$\begin{aligned} \hat{w}_t^i = \frac{C_{i,t} + \alpha - 1}{t + \alpha + \beta - 2} \end{aligned}$$

where $C_{i,t}$ is the number of correct predictions made by participant *i* before time slice *t*. $\alpha , \beta$ are the prior parameters of the Beta distribution, typically set to $\alpha = \beta = 1$.

Estimate the true labels using a weighted voting mechanism based on participant quality:

3 $$\begin{aligned} \hat{y}_t = \arg \max _k \sum _{i=1}^M w_t^i \cdot \mathbb {I}(x_{i,t} = k) \end{aligned}$$

where $\mathbb {I}(\cdot )$ is an indicator function, which takes the value 1 when participant *i*’s prediction equals class *k*, and 0 otherwise.

This algorithm has significant advantages in online processing efficiency. Based on dynamic system modeling, it inherently supports online label aggregation without the need to store historical data. It completes quality estimation in a single pass, avoiding the multiple rounds of iteration required by traditional methods.

### Reward allocation based on participant contributions and peer consistency

Based on an accurate evaluating of participant contributions, it is essential to further design a fair reward allocation mechanism. We propose the Weighted Peer Truth Serum (WPTS) algorithm based on the PTSFD algorithm proposed by Witt et. al.^27^. This algorithm not only accurately allocates rewards, but also demonstrates stronger robustness in the face of collusion attacks. The core idea of the algorithm is to construct an incentive mechanism grounded in Bayesian Nash equilibrium theory:

- Use the participant quality $w_i$ estimated in the previous section for weighting;
- Reward participants when their predictions are consistent with peer consensus;
- Design a penalty mechanism that penalizes deviations from the consensus to maintain system stability.

When the predictions submitted by participants are consistent with peer consensus, their reward is inversely proportional to the normalized support of the consensus, i.e., $\tau _0 = 1/(R_j(x_{ij}) - \beta )$, exponentially amplifying the benefits of a few high-quality participants. This design strictly adheres to incentive compatibility constraints and can only achieve maximum expected returns when participants continue to adopt honest strategies.

During peer selection, the algorithm performs a weighted random sampling based on the participant quality estimates $w_i$, selecting *k* peer participants:

4 $$\begin{aligned} P_{\text {select}}(j) = \frac{w_j}{\sum _{l=1}^M w_l} \end{aligned}$$

For the selected peer participants, a quality-weighted voting scheme is used to generate the consensus label:

5 $$\begin{aligned} x_{\text {consensus}} = \arg \max _k \sum _{j \in P} w_j \cdot \mathbb {I}(x_j = k) \end{aligned}$$

where *P* represents the selected set of peers.

Finally, the reward calculation formula for participant *i* is:

6 $$\begin{aligned} \tau _i = \lambda \cdot w_i \cdot \left( \frac{1}{R_j(x_i)} - \beta \right) \end{aligned}$$

where $\lambda$ is a reward scaling factor that controls the global reward level. $w_i$ is the quality estimate of participant *i* (from the label aggregation algorithm). $R_j(x_i)$ is the normalized support for predicting $x_i$. $\beta$ is a penalty parameter that determines the strength of punishment for deviating from the consensus.

**Algorithm 1:**
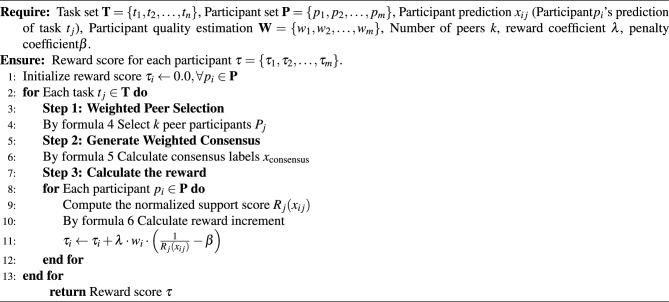
WPTS algorithm

Through a carefully designed reward function, the honest strategy is ensured to be the optimal choice for participants. When a participant deviates from honesty, the penalty term $\beta$ dominates the reward decay, thereby preserving system stability. Moreover, the quality-weighted peer selection mechanism makes it more likely for high-quality participants to be chosen as peers, reducing the risk that malicious nodes manipulate the consensus through numerical superiority.

### Reputation computation based on the extended subjective logic model

Building a long-term reputation mechanism based on contribution evaluation and reward allocation is crucial for maintaining the stability of FL systems. The reputation system can not only record the historical performance of participants, but also provide reference for future task allocation and partner selection.

We adopt an extended Subjective Logic model^45^ to construct the reputation computation mechanism. Subjective Logic, as a probabilistic reasoning framework, can effectively handles trust evaluation under uncertainty. By incorporating a time-decay factor and multi-dimensional evaluation metrics, we extend traditional Subjective Logic into a dynamic reputation model tailored for FL scenarios.

In the FL environment, the reputation evaluation of task publisher *i* toward participant *j* can be represented by a Subjective Logic opinion triplet:

7 $$\begin{aligned} \omega _{i \rightarrow j} = \{b_{i \rightarrow j}, d_{i \rightarrow j}, u_{i \rightarrow j}\} \end{aligned}$$

where $b_{i \rightarrow j}$ is the trustworthiness, representing node *i*’s positive evaluation of participant *j*. $d_{i \rightarrow j}$ is the distrust, representing node *i*’s negative evaluation of participant *j*. $u_{i \rightarrow j}$ is the uncertainty, indicating the degree of indeterminacy in the evaluation. These components satisfy the constraint:

$$b_{i \rightarrow j} + d_{i \rightarrow j} + u_{i \rightarrow j} = 1$$

Based on historical interaction data, the opinion triplet is computed as follows:

8 $$\begin{aligned} {\left\{ \begin{array}{ll} b_{i \rightarrow j} = (1 - u_{i \rightarrow j}) \frac{p_{ij}}{p_{ij} + n_{ij}} \\ d_{i \rightarrow j} = (1 - u_{i \rightarrow j}) \frac{n_{ij}}{p_{ij} + n_{ij}} \\ u_{i \rightarrow j} = 1 - q_{i \rightarrow j} \end{array}\right. } \end{aligned}$$

Where $p_{ij}$ is the number of positive interactions between node *i* and participant *j*. $n_{ij}$ is the number of negative interactions between node *i* and participant *j*. $q_{i \rightarrow j}$ is the communication quality, representing the probability of successful data transmission. The final reputation score of participant *j* is computed as:

9 $$\begin{aligned} R_{i \rightarrow j} = b_{i \rightarrow j} + \alpha u_{i \rightarrow j} \end{aligned}$$

where $\alpha \in [0,1]$ is the uncertainty weight parameter.

Considering the timeliness of reputation evaluations, a time-decay factor *D* is introduced to dynamically adjust the influence weight of historical evaluations:

10 $$\begin{aligned} D = e^{-\lambda (t-t_r)} \end{aligned}$$

Where *t* is the current time. $t_r$ is the time of the last reputation computation. $\lambda$ is the decay adjustment factor that controls the rate of decay. By applying this time-decay mechanism, the reputation system can more accurately reflect participants’ recent behavioral patterns and trustworthiness.

Integrating the output of the aforementioned algorithm, we design a multi-dimensional reputation evaluation mechanism that jointly considers the following three dimensions:

1. The data quality dimension $Q_j$, directly using the output result of the label aggregation algorithm:
  11 $$\begin{aligned} Q_j = w_j^T \end{aligned}$$
  where $w_j^T$ is the quality evaluation of participant *j* at the final time slice *T*.
2. Participation activity $A_j$, based on the participant’s task completion record:
  12 $$\begin{aligned} A_j = \frac{1}{Z} \sum _{z \in Z} h_z \end{aligned}$$
  Where *Z* is the total number of tasks in the evaluation period, and $h_z$ is an indicator variable denoting whether participant completed task *z*.
3. Behavioral stability $S_j$, measuring the consistency of participants’ behavior through the variance of quality evaluation
  13 $$\begin{aligned} S_j = 1 - \frac{\sum _{t=1}^{T} (w_j^t - \bar{w_j})^2}{T} \end{aligned}$$
  Where $\bar{w_j}$ is the average quality evaluation of participant *j*.

To fully leverage the evaluation information from other nodes in the network, an indirect reputation aggregation mechanism is designed. Use improved cosine similarity to evaluate the similarity between nodes:

14 $$\begin{aligned} \text {Sim}(i, x) = \frac{\sum _{j \in N_x} (R_{x \rightarrow j} - \bar{R_x})(R_{i \rightarrow j} - \bar{R_i})}{\sqrt{\sum _{j \in N_x} (R_{x \rightarrow j} - \bar{R_x})^2} \sqrt{\sum _{j \in N_x} (R_{i \rightarrow j} - \bar{R_i})^2}} \end{aligned}$$

where $N_x$ represents the set of participants evaluated jointly by nodes *i* and *x*.

Indirect reputation evaluation are aggregated based on similarity weights:

15 $$\begin{aligned} R_{i \rightarrow j}^{\text {indirect}} = \sum _{x \in N} \text {Sim}(i,x) \cdot R_{x \rightarrow j} \cdot R_x \end{aligned}$$

Finally, the direct and indirect reputations are fused through weighted integration.

16 $$\begin{aligned} R_{i \rightarrow j}^{\text {final}} = \gamma R_{i \rightarrow j}^{\text {direct}} + (1-\gamma ) R_{i \rightarrow j}^{\text {indirect}} \end{aligned}$$

Where $\gamma \in [0,1]$ is the weight parameter for direct reputation.

The proposed reputation computation mechanism forms a complete closed-loop system with the aforementioned algorithms:

- Receive the quality assessment results from the label aggregation algorithm.
- Update interaction records based on participants’ quality assessments and behavioral history.
- Output reputation scores to guide participant selection and task assignment in the next round.

The three core algorithms of IMFLKD form a processing framework, with label aggregation algorithm outputting quality assessment, WPTS algorithm allocating rewards based on quality assessment, and reputation calculation algorithm updating participant reputation based on quality assessment and participation behavior, thereby incentivizing long-term honest participation. The overall time complexity is controlled within the range of O(MT+nk+M), and the space complexity is O(M).

## Results and performance analysis

The experiments primarily include algorithm effectiveness validation and robustness testing. Our approach is compared against the following blockchain-enabled FL incentive algorithms:

BFL^45^: A FL framework that introduces a reputation mechanism to assess the reliability of mobile devices. It employs a multi-weight Subjective Logic model to dynamically evaluate participants and integrates blockchain technology to achieve decentralized reputation management, ensuring the immutability of records.

FREB^46^: A FL framework that deeply integrates a reputation evaluation mechanism with blockchain technology. Transparency is guaranteed through smart contracts, and a comprehensive evaluation method combining a multi-weight Subjective Logic model with the Shapley value is adopted.

PTSFD^27^: A peer truth serum mechanism for federated distillation that constructs an incentive ecosystem by implicitly comparing participant contributions, enabling precise identification and rewarding of honest behavior.

### Performance validation of label aggregation algorithm

To evaluate the performance of our proposed label-aggregation-based model quality assessment algorithm, we simulated a FL environment with 10 participating nodes. Each node used 24,000 samples from the EMNIST dataset as its local training set. Additionally, 40,000 samples from the MNIST dataset were employed for KD.

**Fig. 3:**
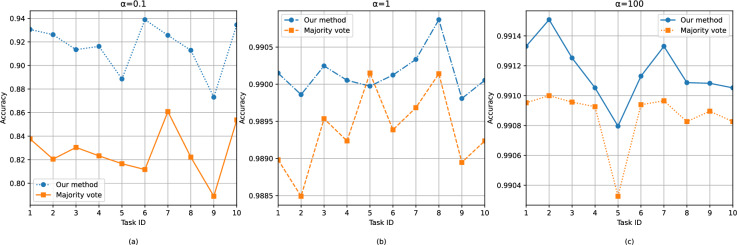
Accuracy of label aggregation algorithm under different $\alpha$ parameters.

We collected the accuracy performance of the proposed method and majority voting across multiple tasks under different Dirichlet distribution parameters $\alpha$. As shown in Fig. 3, when $\alpha = 0.1$, our method achieved a significant improvement over majority voting, indicating that it better handles noise and uncertainty under highly non-IID (heterogeneous) data distributions. At $\alpha = 1$ and $\alpha = 100$, our approach still maintained higher accuracy with smaller fluctuations, demonstrating superior stability.

This demonstrates that our method can effectively estimate true labels when addressing the label aggregation problem in KD-FL, exhibiting robust performance even under non-uniform data distributions and in the presence of noise. Moreover, since the algorithm is based on dynamic system modeling, it inherently supports online label aggregation–enabling real-time updates of worker quality and true labels without storing historical data, thereby achieving strong performance without increasing time or space complexity.

We evaluated the runtime of the algorithms under a distillation dataset size of 40,000, with results shown in Table 1. The algorithm proposed exhibits runtime comparable to that of majority voting^47^, demonstrating high computational efficiency. PTSFD^27^ improves performance by introducing peer comparison, but this comes at the cost of increased algorithmic complexity. Although GTG-Shapley^35^ accelerates the convergence of Shapley values by leveraging its guided sampling technique to effectively prune unnecessary submodel generation and utility evaluations, it inherently requires reconstructing submodels to assess each participant’s contribution, resulting in significantly higher computational overhead compared to other methods.

**Table 1:** The impact of the number of clients on computational cost.

N	Majority voting^47^	Our method	PTSFD^27^	GTG-Shapley^35^
5	0.1276s	0.2232s	6.8415s	2564.49s
6	0.1524s	0.2676s	8.2098s	3010.65s
7	0.1788s	0.3122s	9.5781s	3523.53s
8	0.2032s	0.3586s	10.8694s	4157.85s
9	0.2286s	0.4014s	12.3147s	4549.55s
10	0.2543s	0.4465s	13.6833s	5062.17s

In terms of computational complexity, our algorithm maintains a linear complexity of O(TN), where T denotes the number of participating client nodes and N denotes the size of the distillation dataset. While preserving the same time complexity (O(TN)) and space complexity (O(T+N)) as majority voting, our method achieves enhanced functionality in estimating participant contributions. When evaluated on a distillation dataset of size 40,000 with 10 clients, it still achieves low runtime (0.446 seconds for label aggregation), demonstrating its suitability for resource-constrained blockchain environments–particularly crucial when handling large-scale datasets and a large number of clients.

To evaluate the robustness of our algorithm against malicious attacks, we conducted a label-flipping attack experiment in a FL setting. Label-flipping attack is a common form of data poisoning, wherein an adversary manipulates data labels to disrupt the model training process, thereby degrading the model’s accuracy and reliability. We scale the number of participating clients to 100, training ResNet-18 on CIFAR-10 dataset, and designate clients 1 through 30 as malicious attackers that flip the labels of their local training data to simulate a label-flipping attack.

**Fig. 4:**
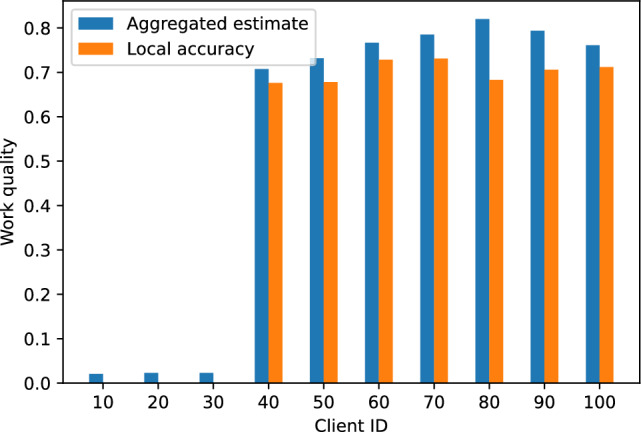
Comparison between aggregated estimates and local accuracy.

**Fig. 5:**
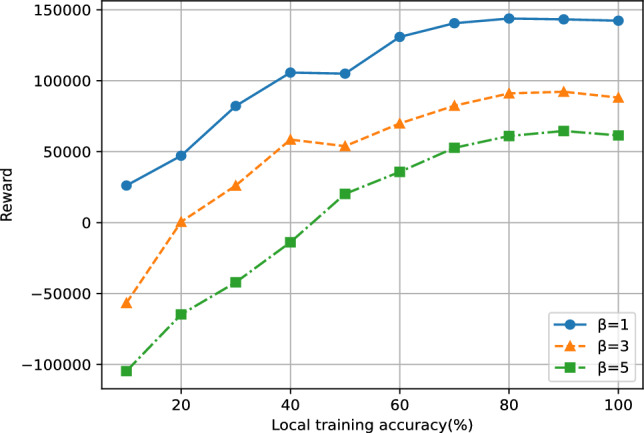
The impact of local training accuracy on rewards.

As shown in Fig. 4, it can be observed that under a label-flipping attack, the local accuracy of the affected clients drops dramatically to approximately 0.01%. This severe performance degradation indicates that the attack substantially disrupts the model learning process. Notably, however, the model quality estimates for these clients–produced by our proposed label aggregation method–are close to 0%. This demonstrates that our label aggregation algorithm, by integrating information from multiple data sources, enhances robustness against unreliable or malicious participants and effectively identifies nodes compromised by adversarial attacks. This capability is crucial in FL environments.

### Fairness of incentive mechanism and verification against collusion

To simulate the heterogeneity of a FL environment, we assigned 100 clients customized early-stopping criteria using the CIFAR-10 dataset. This strategy reflects the varying levels of effort exerted by each client during training, where a higher local training accuracy is treated as an indicator of greater effort and is typically associated with more substantial rewards. Furthermore, by adjusting the $\beta$ parameter, we can flexibly modulate the penalty imposed on incorrect predictions, thereby enabling fine-grained control over the incentive mechanism, as shown in Fig. 5. Additionally, to deter potential malicious behavior, we introduced an initial deposit mechanism: clients engaging in malicious activities risk forfeiting their deposit. This design not only enhances system security but also fosters trust within the FL community, encouraging honest and responsible participation.

To simulate collusion attacks that may occur in a FL environment, we model the attack behavior by defining a conditional function, where the reported label $LR_i$ is determined based on the value of the evaluation label $LE_i$. The expression of this strategy is as follows:

$$LR_i = {\left\{ \begin{array}{ll} 1 & \text {if } LE_i \in \{1,2,3,4,5\} \\ 0 & \text {if } LE_i \in \{6,7,8,9,0\} \end{array}\right. }$$

**Fig. 6:**
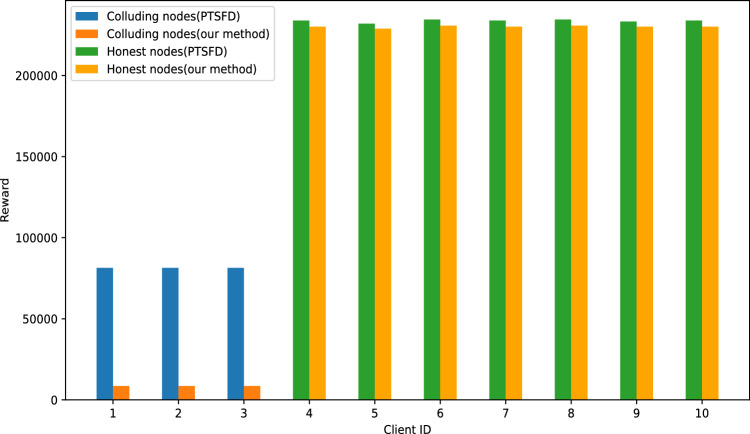
Reward comparison under collusion attacks.

**Fig. 7:**
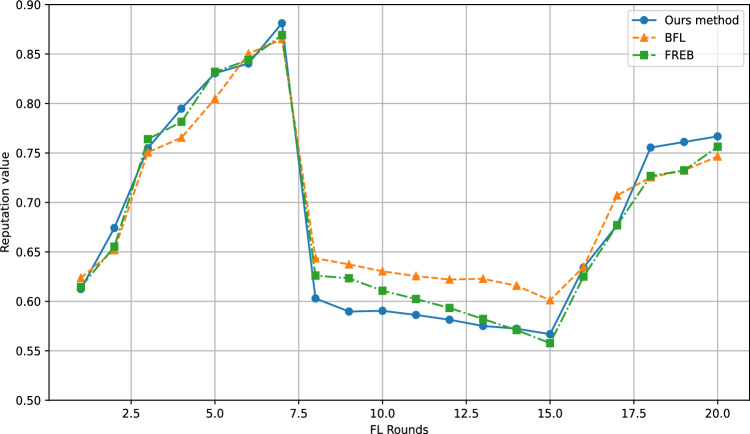
Reputation changes of different methods.

As shown in Fig. 6, In the PTSFD method, the system indiscriminately assigns high rewards to low-frequency answers. As a result, even colluding nodes can still obtain substantial rewards, inadvertently incentivizing low-quality or malicious behavior. In contrast, our approach uses the aggregated estimate of each client’s model quality as a weighting factor for rewards. Consequently, even if a node provides a low-frequency answer, it can still receive a high reward if its model quality is high. Conversely, nodes with low model quality cannot earn significant rewards, regardless of how infrequent their answers are. This design significantly reduces the rewards attainable by colluding nodes, thereby effectively deterring malicious behavior.

### Validity verification of reputation mechanism

To validate the effectiveness of our method in evaluating the reputation of malicious participants, we configured a scenario in which malicious participants behaved benignly during the first seven task rounds, thereby accumulating a relatively high reputation score. Starting from the eighth to the fifteenth task rounds, they switched to adversarial behavior–specifically, uploading datasets with flipped labels and submitting soft labels derived from models trained on this tampered data. This setup aims to simulate a realistic attack pattern where malicious actors, after gaining sufficient trust, begin engaging in destructive activities, enabling us to observe and analyze the corresponding changes in their reputation scores.

The experimental results are shown in Fig. 7. During the first seven rounds, our approach yielded reputation scores for attackers similar to those of BFL and FREB, with no significant differences observed, and the reputation values generally exhibited an upward trend across all methods. However, when the attackers launched their attacks from rounds 8 to 15, their reputation scores dropped noticeably. Compared to the other two methods, our proposed scheme incorporates the weights of the final aggregated model when evaluating contributions, which demonstrates clear advantages in the presence of negative interactions. Specifically, the reputation scores of attackers declined more rapidly under our method, indicating that it can more effectively identify historical malicious behavior and assign lower evaluation scores accordingly.

## Conclusion and future work

This paper addresses the limitations of traditional FL systems, particularly their reliance on centralized architectures and inadequate data privacy protection, by proposing a blockchain-enabled decentralized KD-FL framework. To tackle the core challenge of insufficient incentive mechanisms within this framework, we introduce IMFLKD, a fair incentive mechanism for blockchain-enabled FL based on knowledge distillation. The IMFLKD mechanism first proposes a contribution evaluation algorithm tailored for label aggregation in KD-FL scenarios. This algorithm employs a dynamic Bayesian network to jointly estimate participant quality and ground-truth labels in an online manner, with a time complexity of O(MT). It inherently supports online processing and requires no storage of historical data. Second, we design an incentive-compatible reward allocation algorithm grounded in Bayesian game equilibrium. By integrating the quality assessment from label aggregation with an enhanced peer truth serum, the algorithm leverages quality-weighted peer selection and consensus generation to resist collusion attacks and ensure fairness in reward distribution. Third, we construct a multi-dimensional reputation evaluation mechanism based on extended Subjective Logic, which comprehensively considers factors such as data quality, participation activity, and behavioral stability. A time-decay factor is introduced to dynamically adjust the influence weight of historical evaluations, and an indirect reputation aggregation mechanism enables cross-node trust propagation.

Experimental results demonstrate that our proposed blockchain-enabled decentralized KD-FL significantly reduces communication and storage overhead while maintaining model accuracy. The IMFLKD incentive mechanism improves label aggregation accuracy by approximately 10% compared to majority voting methods and exhibits superior performance in terms of time efficiency and robustness against attacks, thereby validating its practicality and reliability in complex FL environments.

In future work, we plan to further refine the incentive mechanism by incorporating additional evaluation dimensions, such as data diversity, model innovativeness, and network stability, to more holistically quantify participant contributions, and to design more granular and personalized reward allocation strategies.

## Data Availability

The datasets used in this work – MNIST and EMNIST are publicly available at the following URL: http://yann.lecun.com/exdb/mnist/, https://www.nist.gov/itl/products-and-services/emnist-dataset. CIFAR10 is publicly available at the following URL: https://www.cs.toronto.edu/ kriz/cifar.html
